# Application of the “telescopic rod” in a combined surgical technique for the treatment of congenital pseudarthrosis of the tibia in children

**DOI:** 10.1186/s13018-021-02649-2

**Published:** 2021-08-26

**Authors:** Yaoxi Liu, Ge Yang, Guanghui Zhu, Qian Tan, Jiangyan Wu, Kun Liu, Jin Tang, Haibo Mei

**Affiliations:** Department of Pediatric Orthopaedics, Hunan Children’s Hospital, The Pediatric Academy of University of South China, No 86 Ziyuan Road, Yuhua District, Changsha City, 410007 Hunan Province China

**Keywords:** Children, Congenital pseudarthrosis of tibia, Telescopic rod, Initial effect

## Abstract

**Background:**

The current surgical treatment of choice is the combination surgical technique, involving tibial intramedullary fixation to maintain the mechanical axis and mechanical stability of tibial pseudarthrosis. In traditional combined surgery, the Williams rod is often used. Long-term intramedullary fixation of the foot and ankle will affect the ankle joint function of children. The intramedullary rod is relatively shorter due to the growth of the distal tibia. In addition, there are some complications such as epiphyseal bone bridge and high-arched foot. The use of a telescopic intramedullary rod may avoid these complications.

**Purposes:**

To investigate the initial effect of the “telescopic rod” in a combined surgical technique for the treatment of congenital pseudarthrosis of the tibia in children.

**Methods:**

A retrospective study including 15 patients with Crawford type IV CPT who were treated using a combined surgical technique and the telescopic rod from January 2017 to May 2018. The average age at the time of surgery was 43.3 months (16–126 months). Of the 15 patients, 7 had proximal tibia dysplasia and 12 exhibited neurofibromatosis type 1. The combined surgical technique using the telescopic rod included the excision of pseudarthrosis, intramedullary rod insertion, installation of Ilizarov’s fixator, tibia-fibular cross union, and wrapping autogenic iliac bone graft. The incidence of refracture, ankle valgus, tibial valgus, and limb length discrepancy (LLD) in patients were investigated.

**Results:**

All patients achieved primary union with an average follow-up time of 37.3 months (26–42 months). The mean primary union time was 4.5 months (4.0–5.6 months). Nine cases showed LLD (60%), with an average limb length of 1.1 cm (0.5–2.0 cm). Ankle valgus, proximal tibial valgus, telescopic rod displacement, and epiphyseal plate tethering occurred in 1 case (6.6%) (18°), 3 cases (20%) (10°, 5°, and 6°, respectively), 6 cases (40%), and 2 cases (13%), respectively. There were no refractures during the follow-up periods.

**Conclusion:**

Although there are complications such as intramedullary rod displacement while using the telescopic rod in a combined surgery, the primary healing rate of congenital pseudarthrosis of the tibia in children is high.

## Introduction

Congenital pseudarthrosis of the tibia (CPT) is one of the most challenging diseases in children’s orthopedics [1–6]. At present, surgical treatment often advocates the combined surgical technique involving tibial intramedullary fixation to maintain the mechanical axis and mechanical stability of pseudarthrosis. Besides, the major advancement in CPT surgery of recent years is the cross-union technique. Maximizing the tibial cross-sectional area of the healed segment could reduce the rate of refracture in CPT [6]. In traditional combined surgery, the Williams rod is often used. Long-term intramedullary fixation of the foot and ankle will affect the ankle joint function of children. The intramedullary rod is relatively short due to the growth of the distal tibia. In addition, there are some complications such as the epiphyseal bone bridge and high-arched foot. In order to avoid these complications, we used the “telescopic intramedullary rod” in a combined surgery to treat children with CPT to maintain a good mechanical axis of the tibia. This should not affect the ankle joint function. Consequently, the tibia can be protected by the intramedullary rod over time to prevent refracture. In this investigation, we analyzed the clinical data of the children who were treated with the telescopic intramedullary rod. We evaluated the incidence of primary healing in children with congenital pseudarthrosis of the tibia and identified the complications, such as unequal tibia length and ankle valgus.

## Patients and methods

Between January 2017 and May 2018, 15 cases of congenital pseudarthrosis of the tibia were included in the investigation. There were 12 males and 3 females. There were 7 affected tibias on the left and 8 on the right. The average age at the time of surgery was 43.3 months (16–126 months). There were 7 cases with proximal tibial dysplasia and 12 cases with neurofibromatosis type 1. Three patients had a family history of neurofibromatosis type 1. This investigation was approved by the ethics committee of the hospital. The inclusion criteria were (1) congenital pseudarthrosis of the tibia with Crawford type IV, (2) there are no multiple angular deformities of the tibia, and (3) all operations performed or supervised by a single surgeon. Cases of traumatic or infectious pseudarthrosis of the tibia were excluded.

### Surgical technique

Surgery for the left side was performed as follows. The patient was placed in a supine position after anesthesia. The left thigh was attached with a positive pressure tourniquet. The first incision, measuring approximately 6.0 cm in length, was made at the outer edge of the right iliac crest. The outer plate of the iliac bone was exposed under the periosteum. A rectangular section of the cortex was obtained from the outer table of the ilium, and most of the cancellous bone, which was obtained, was curetted from the supra-acetabular region while keeping the inner wall intact. Subsequently, a series of holes were made in the rectangular cortex using a fine Kirschner wire and absorbable sutures were weaved into the holes to obtain a cylindrical shape for wrapping the graft.

A 3.5-cm incision was made at the proximal fibular side of the left leg. The fibula was osteotomized to prevent a high tension resulting from the fusion of the tibia and the fibula and to avoid the curving of the fibula when the tibia and fibula fuse. Osteotomization can also lead to better fusion. The anterior, lateral, and posterior compartments of the tibial pseudoarthrosis and the middle and distal fibula were exposed. The thickened periosteum around the pseudarthrosis of the tibia was removed. A telescopic rod was implanted as per the pre-operation planning.

Two rings (Ilizarov’s fixator) were placed in the proximal and distal tibia. The quadrilateral ilium cortex was used to wrap around the pseudarthrosis. The cancellous bone of the ilium was filled between the tibia and fibula. No.1 suture was tightened and tied to fix the graft site and the incisions were sutured.

### Post-operation management

The nurse cleaned the needle path once every 2 days with normal saline. We monitored the patients for the clinical manifestation of osteofascial compartment syndrome. Patients underwent radiographic examination every 2 months. When the pseudarthrosis of the tibia had consolidated, Ilizarov’s fixator was removed and a long leg cast (a tube-type cast) was applied for about 2 months. After the cast was removed, a protective long leg knee-ankle-foot brace was used to protect the involved extremity during weight-bearing and walking. The brace was worn most of the time, including sleeping and swimming, until skeletal maturity was attained. The only time the brace was removed was for bathing.

### Clinical and radiographic evaluation methods

1. Criteria for the healing of CPT: A RUST score of more than 8 points indicated initial healing [7].
2. The length of the tibia, that is, the distance from the midpoint of the proximal epiphyseal plate to the midpoint of the distal epiphyseal plate, was measured using a picture archiving and communication systems (PACS).
3. If the radiographic films showed discontinuity of the bone cortex, it was considered that refracture has occurred.
4. Measurement of the tibial valgus angle. PACS was used to measure the angle between the epiphyseal line of the tibial proximal axis and the anatomic axis of the tibia. If the valgus of the tibial proximal axis was more than 3°, it was defined as tibial valgus.
5. The angle between the anatomic axis of the distal third of the tibia and the tibiotalar joint surface was measured by PACS. If the angle was greater than 5°, it was defined as ankle valgus.

## Results

**Fig. 1:**
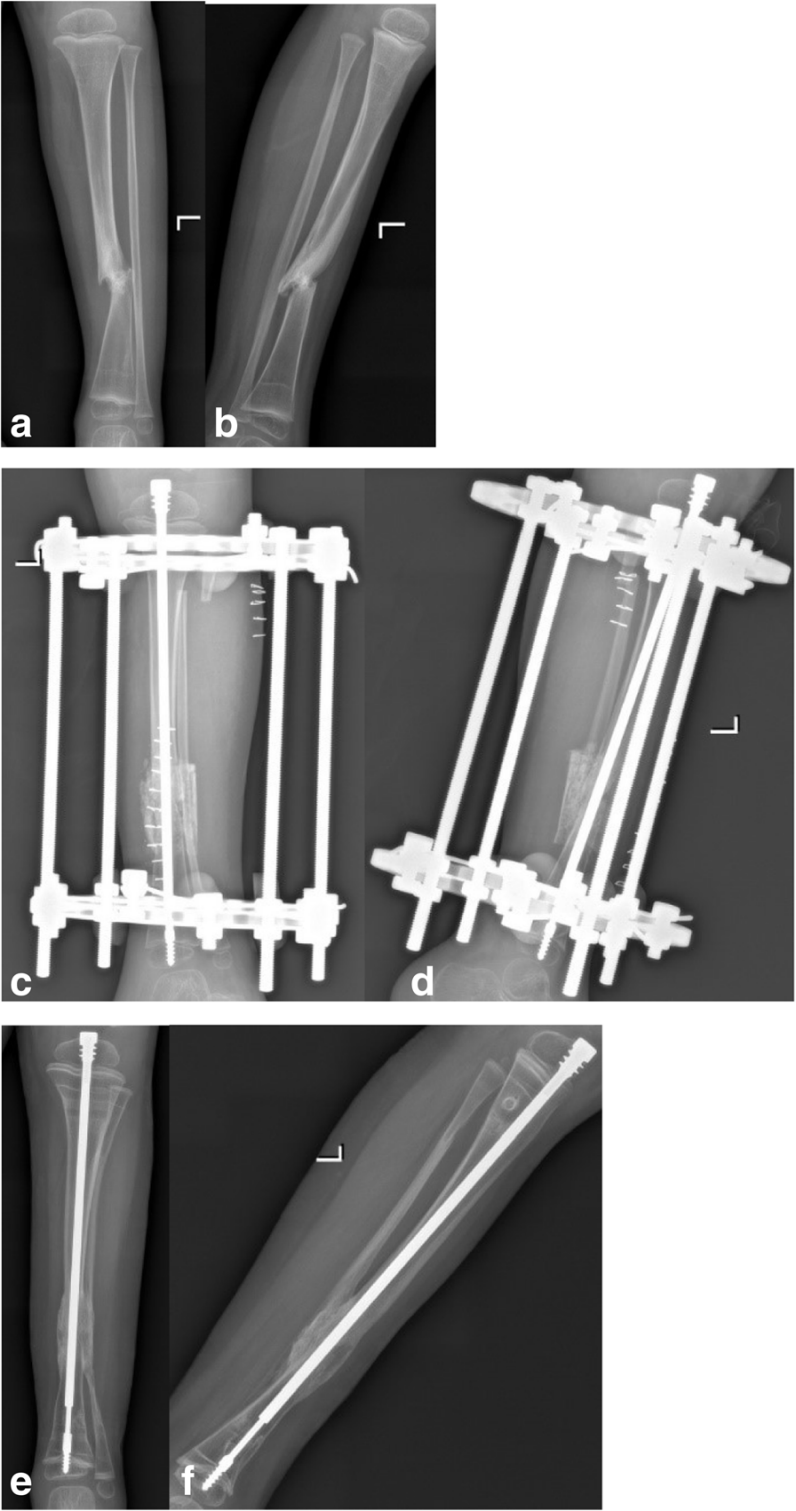
**a**, **b** A 4-year-old boy. Preoperative anteroposterior and lateral radiographs. **c**, **d** The X-ray image at 1 week post-operation. **e**, **f** Twelve months after the operation

**Fig. 2:**
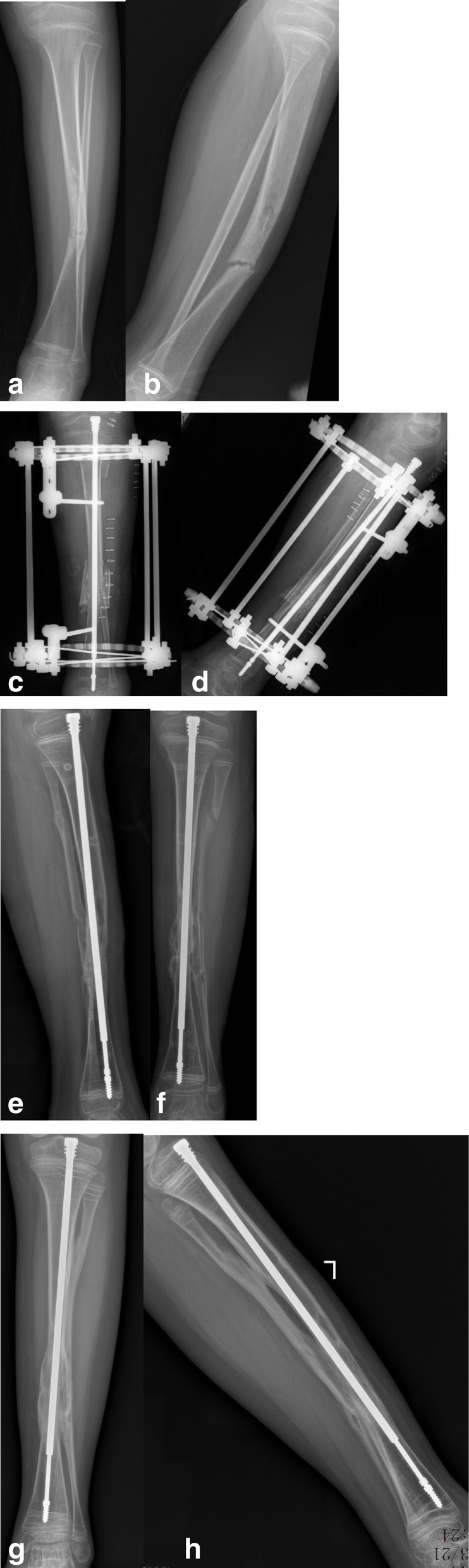
**a**, **b** A 6-year-old girl. Preoperative anteroposterior and lateral radiographs. **c**, **d** The X-ray image at 1 week of post-operation. **e**, **f** Seven months after the operation. **g**, **h** Sixteen months after the operation with telescopic rod displacement

**Fig. 3:**
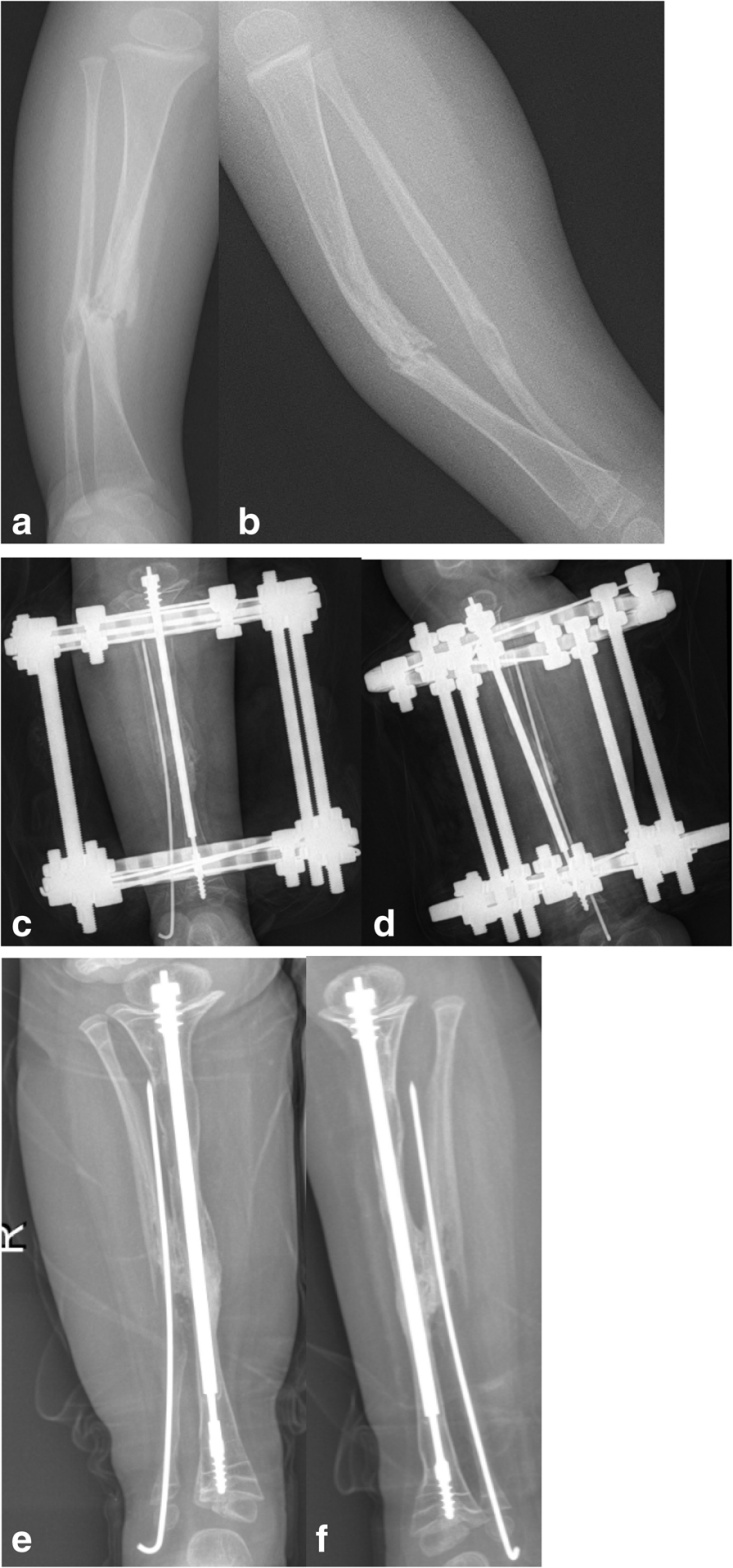
**a**, **b** A 2-year-old boy. Preoperative anteroposterior and lateral radiographs. **c**, **d** Four months post-operation. **e**, **f** Seven months post-operation with telescopic rod displacement and epiphyseal plate tethering

All patients achieved primary union with an average follow-up time of 37.3 months (26–42 months) (Fig. 1). The average primary union time was 4.5 months (4.0–5.6 months). Nine cases showed LLD (60%), with an average limb length of 1.1 cm (0.5–2.0 cm). Ankle valgus occurred in 1 case (18°, 6.6%), proximal tibial valgus occurred in 3 cases (20%) (10°, 5°, and 6°, respectively), telescopic rod displacement occurred in 6 cases (40%) (Fig. 2), and the epiphyseal plate tethered in 2 cases (13%) (Fig. 3). There were no refractures during the follow-up periods. There were 15 cases with normal movement and function of the ankle joint with an average dorsiflexion 24° (20~30°) and with an average plantar flexion 43° (40~50°). One case of ankle valgus was treated using the epiphyseal block with a distal tibial screw. There were also three cases with proximal tibial valgus and three cases with proximal tibial 8 plate block. The deformities were corrected for these patients (typical case: Fig. 1a–e).

### Patient characteristics

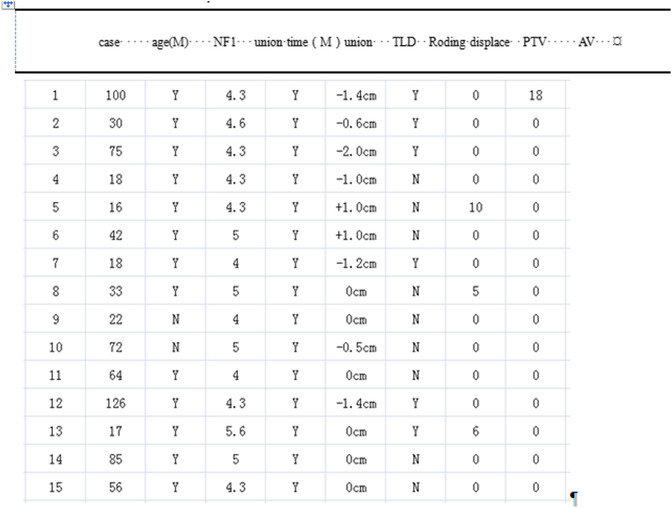



*NF1* neurofibromatosis type 1, *TLD* tibial length discrepancy, *AV* ankle valgus, *PTV* proximal tibial valgus. “+” stands for overgrowth, and “−” stands for shortening

## Discussion

Presently, the primary union rate of CPT is relatively high. However, preventing complications is challenging. Refracture is the most serious complication in CPT patients following the achievement of primary union, and a small cross-sectional area of the pseudarthrosis is a risk factor for refracture. However, 3-in-1 and 4-in-1 bone osteosynthesis were applied along with combined surgery to increase the cross-sectional area of the healed segment thereby [5, 6]. These surgical methods can reduce the incidence of refracture. Besides, the telescopic intramedullary rod attached is of great importance to the treatment of CPT.

### Advantages and disadvantages of traditional intramedullary rods

The traditional intramedullary rod, also known as the Williams rod, has the advantages of being able to fix the ankle joint, extending the fixed arm of force, and making the fixation of the tibial pseudarthrosis reliable. The disadvantages include the following: the ankle joint is prone to be stiff after long-term fixation, which affects the ankle joint function and may even affect the development of the talus, the formation of high heels, and other complications. The bridge of the epiphyseal plate is easily formed by the Williams rod repeatedly passing through the epiphyseal plate. We reviewed the literature on the treatment of CPT using intramedullary rods. In 2000, the European Paediatric Orthopedics Society conducted a multicenter study on 340 cases of CPT. The results showed that the initial healing rate of CPT was 75%, and the intramedullary rod was recommended to prevent refractures [8]. El-Rosasy et al. [9] reported 17 children with CPT, with an average age of 8 years. All children achieved initial healing of the pseudarthrosis of the tibia. Half of the patients were followed up until the bone matured. The incidence of refracture was 68% when using the Ilizarov external fixation device alone. However, the incidence of refracture decreased to 29% after using the Ilizarov external fixation device and the intramedullary rod [9]. Johnston [10] reported 23 cases of CPT treated by bone grafting and intramedullary rod fixation, with an average age of 2 years and 4 months and an average follow-up time of 9 years. The initial healing rate was 87%, and 13% of the cases had persistent non-union. The researchers pointed out that the use of intramedullary rods can achieve healing of the tibial pseudarthrosis, prevent refracture, and maintain a good mechanical axis [10]. In 2004, Dobbs et al. [11] reported that CPT patients were treated by intramedullary rod fixation of the foot and the ankle and autogenous iliac bone transplantation. It was considered that refracture, displacement of the intramedullary rod, or relative shortening of the intramedullary rod due to the growth of the distal tibia, valgus of the ankle, and unequal length of the limbs after pseudarthrosis of the tibia were common problems in the treatment of CPT. Most of these issues resolved satisfactorily after surgical treatment. In 2008, Thabet et al. [12] reported that the resection of the diseased periosteum combined with free periosteum transplantation, bone transplantation, tibial intramedullary rod, and Ilizarov external fixator can improve the fracture healing rate and reduce the rate of refracture. It was considered that this operation is an effective treatment method.

At present, the most serious complication is refracture after CPT healing. Prevention of refracture should be one of the main goals of CPT treatment. Retention of intramedullary rod fixation may be one of the effective measures to prevent refracture. After the tibial pseudarthrosis is healed, it is generally necessary to adjust the position of the intramedullary rod to restore the ankle joint activities. Removal of the intramedullary rod after the tibial pseudarthrosis is healed is not recommended [13, 14]. Ankle fixation generally does not exceed 2 years, and ankle fixation less than 2 years has little impact on ankle functions.

### The original intention of using telescopic intramedullary rods

The advantage of the telescopic intramedullary rod in the treatment of CPT is that the ankle joint does not need fixation, and the effect of the operation on ankle joint function can be avoided. The intramedullary rod can slide with the growth of the tibia. The tibia is always protected by the intramedullary rod, which may prevent the occurrence of refracture. Therefore, the use of telescopic intramedullary rods for CPT is the current trend of standardized surgical treatment. In 2012, Paley [15] treated 15 children (average age of 4 years) with CPT by periosteal transplantation, autogenous cancellous bone graft, tibial telescopic intramedullary rod, external fixation, tibiofibular fusion, bisphosphonates, and bone morphogenetic proteins. After an average follow-up of 2 years, all patients achieved union. There were no refractures [15].

### Key points for the operation of the telescopic intramedullary rod

(**1)** To avoid the influence of the thick thread on the growth of the epiphyseal plate, the outer sleeve thread cannot be screwed into the proximal epiphyseal plate of the tibia. With the growth of the tibia, the distal end of the core pin may move to the proximal end. Therefore, when the distal end of the core pin is screwed into the epiphysis of the distal tibia, it should be positioned as close to the ankle joint surface as possible, but the movement of the ankle joint should be checked to avoid the influence of the core on ankle joint functions. (**2)** The knee joint ligament injury should be avoided as much as possible when the patellar incision is made and the extendable intramedullary rod is placed. (**3)** The cranial and caudal of the Ilizarov rings should be parallel to each other to prevent the inner core from sliding abnormally.

### Problems and process of the operation

With the growth of the tibia, there are problems such as displacement of the intramedullary rod, abnormal sliding of the inner core, and tethering of the proximal epiphyseal plate of the tibia. It may be necessary to replace the telescopic intramedullary rod during growth, because the tibia length of girls is twice that of a 3-year-old, and the length in boys is twice that of a 4-year-old [13]. Therefore, the telescopic intramedullary rod may need to be replaced 1–2 times before the child’s skeleton matures. During the growth period, the child should be closely followed up. In this investigation, there were 6 cases of telescopic intramedullary rod displacement, including 4 cases with the distal end of the intramedullary rod withdrawing from the epiphysis of the distal tibia and 2 cases with the proximal of the intramedullary rod displacement to the distal end. The epiphyseal plate was tethered in 2 cases, and the intramedullary rods were removed at 20 months and 12 months, respectively. In one case, the proximal thread of the telescopic intramedullary rod was screwed into the epiphyseal plate, and the reason for the epiphyseal plate tethering may be related to the abnormal movement of the thread of the telescopic intramedullary rod which entered into the epiphyseal plate. However, the specific reason remains to be further investigated. To prevent the displacement of the telescopic intramedullary rods, it may be necessary to design new telescopic intramedullary rods in the future.

There are some limitations in this investigation, such as a small sample size, a single-center retrospective study design, and a short follow-up time. However, congenital pseudarthrosis of the tibia in children is a rare disease. The preliminary conclusion from the results of this study is that the application of the telescopic intramedullary rod combined with surgery in the treatment of children’s congenital pseudarthrosis of the tibia has a high initial healing rate and facilitates normal ankle functions, but there are complications, such as intramedullary rod displacement.

## Data Availability

All data generated or analyzed during this study are included in this published article.

## Abbreviations

CPT: Congenital pseudarthrosis of the tibia
NF1: Neurofibromatosis type 1
IR: Intramedullary rod
